# Liver Enzyme Elevations in *Plasmodium falciparum* Volunteer Infection Studies: Findings and Recommendations

**DOI:** 10.4269/ajtmh.19-0846

**Published:** 2020-04-20

**Authors:** Mohamed Farouk Chughlay, Samantha Akakpo, Anand Odedra, Katalin Csermak-Renner, Elhadj Djeriou, Cornelis Winnips, Didier Leboulleux, Aditya H. Gaur, G. Dennis Shanks, James McCarthy, Stephan Chalon

**Affiliations:** 1 Medicines for Malaria Venture, Geneva, Switzerland;; 2 QIMR Berghofer Medical Research Institute, Brisbane, Queensland, Australia;; 3 Novartis Pharma AG, Basel, Switzerland;; 4 Sanofi, Gentilly, France;; 5 St. Jude Children’s Research Hospital, Memphis, Tennessee

## Abstract

Malaria volunteer infection studies (VISs) accelerate new drug and vaccine development. In the induced blood-stage malaria (IBSM) model, volunteers are inoculated with erythrocytes infected with *Plasmodium falciparum*. Observations of elevated liver enzymes in the IBSM model with new chemical entities (NCEs) promoted an analysis of available data. Data were reviewed from eight IBSM studies of seven different NCEs, plus two studies with the registered antimalarial piperaquine conducted between June 2013 and January 2017 at QIMR Berghofer, Brisbane, Australia. Alanine aminotransferase (ALT) was elevated (> 2.5 times the upper limit of normal [×ULN]) in 20/114 (17.5%) participants. Of these, 8.9% (10/114) had moderate increases (> 2.5–5 × ULN), noted in seven studies of six different NCEs ± piperaquine or piperaquine alone, and 8.9% (10/114) had severe elevations (> 5 × ULN), occurring in six studies of six different NCEs ± piperaquine. Aspartate aminotransferase (AST) was elevated (> 2.5 × ULN) in 11.4% (13/114) of participants, across six of the 10 studies. Bilirubin was > 2 × ULN in one participant. Published data from other VIS models, using sporozoite inoculation by systemic administration or mosquito feeding, also showed moderate/severe liver enzyme elevations. In conclusion, liver enzyme elevations in IBSM studies are most likely multifactorial and could be caused by the model conditions, that is, malaria infection/parasite density and/or effective parasite clearance, or by participant-specific risk factors, acetaminophen administration, or direct hepatotoxicity of the test drug. We make recommendations that may mitigate the risk of liver enzyme elevations in future VISs and propose measures to assist their interpretation, should they occur.

## INTRODUCTION

*Plasmodium falciparum* volunteer infection studies (VISs), previously referred to as controlled human malaria infection (CHMI) studies, are conducted in non-endemic countries in healthy, malaria-naïve participants. Volunteer infection studies allow pharmacokinetic/pharmacodynamic profiling of new chemical entities (NCEs) to support early go/no-go decisions and enable optimized dose selection for Phase II trials, accelerating drug development.

There are two models of induced *P. falciparum* malaria infection. In the *P. falciparum* sporozoite (*Pf*SPZ) challenge model, sporozoites are inoculated via mosquito bite or by systemic administration (intradermal, intramuscular, or intravenous). This model is generally used to investigate vaccine candidates and chemoprevention therapies, where infection suppression is the primary goal. Consequently, participants are exposed to low-level parasitemia, with early treatment using an appropriate antimalarial. In the induced blood-stage malaria (IBSM) model, malaria-infected erythrocytes are introduced intravenously. Used mainly for investigating NCE antimalarial activity against blood-stage parasitemia, that is, acute malaria treatment, this model has been predominantly conducted at QIMR-Berghofer Medical Research Institute Brisbane, Australia (QIMRB).^1^ To characterize drug parasite killing kinetics, IBSM generates higher parasitemia levels versus *Pf*SPZ challenge, yet typically 1000-fold lower than observed in uncomplicated malaria patients.

This investigation was prompted by observations of elevated alanine aminotransferase (ALT) and aspartate aminotransferase (AST) levels in IBSM studies conducted using several structurally diverse NCEs. Elevated ALT/AST was also observed with piperaquine, a component of the licensed antimalarial dihydroartemisinin–piperaquine, which has no clinical hepatotoxicity. Similar findings across these studies suggested there might be common factors in the IBSM model increasing the risk of transaminase elevation.

Hepatotoxicity is a major reason for drugs to fail clinical development.^2^ Drug-induced liver injury (DILI) provokes liver test abnormalities or liver dysfunction, although diagnosis requires reasonable exclusion of other causes.^3^ Intrinsic DILI is caused when drugs affect hepatocytes, by a variety of dose-dependent mechanisms, and can be predicted in human and animal models.^3^ By contrast, idiosyncratic DILI is rare, unpredictable, and dependent on factors inherent to the individual.^3^ Severe DILI is a leading cause of acute liver failure.^3^ To date, no genetic, metabolic, or other characteristic reliably predicts severe DILI in an individual.^3^ Increases in ALT/AST indicate hepatocellular injury but do not predict progression to severe DILI. Only when liver damage evolves to impair hepatocellular bilirubin excretion and serum bilirubin increases does functional impairment manifest clinically.^3^

The potential for a drug to cause severe DILI is described by Hy’s law, that is, ALT elevation in a participant, usually substantial (≥ 3 times the upper limit of normal [×ULN]), seen concurrently with bilirubin > 2 × ULN.^3^ Hy’s law identifies a drug likely to cause severe DILI (fatal or requiring liver transplant) at a rate roughly 1/10 the rate of Hy’s law cases. Hy’s law is confirmed if the drug caused the liver injury (excluding other causes), if hepatocytes are injured (not biliary obstruction, i.e., alkaline phosphatase [ALP] not substantially elevated), and if the injury was sufficient to impair liver function (hyperbilirubinemia). Finding two Hy’s law cases causally related to drug administration in a clinical program indicates a high potential for severe DILI.^3^ Finding one case requires greater monitoring and investigation.

Understanding the possible risks/consequences for healthy participants enrolled in IBSM Phase I studies is critical, especially when testing NCEs associated with liver safety signals in preclinical species. Also, safety is paramount for new antimalarial drugs and drug combination strategies. Consequently, the underlying mechanism of abnormal ALT/AST findings in IBSM studies and their relevance to drug development programs must be fully considered. This article examines the potential contributions of IBSM study procedures and the tested drugs to ALT/AST elevations in healthy human participants. We also consider recommendations for reducing the risk of ALT/AST elevations in VIS models and aiding their interpretation should they occur.

## METHODS

### Participants and design.

All participants were healthy adults ≥ 18 years old. Data were reviewed from eight studies of seven different NCEs conducted between June 2013 and January 2017 at QIMRB, Brisbane, Australia (Table 1). None of the NCEs had findings in preclinical or clinical studies suggesting hepatotoxic potential at the doses used in the IBSM model. Two studies (QP15C01 and QP15C05) included piperaquine administration to clear asexual parasites, while allowing gametocyte development, for assessment of NCE anti-gametocyte activity. However, in QP15C05, the planned NCE (OZ439) was not administered because ALT/AST levels increased after inoculation and piperaquine treatment. To further understand the potential mechanistic effects of the model on ALT/AST elevations, two additional studies with piperaquine alone were examined: QP13C05 and QP14C21 (Table 1).

**Table 1:** Characteristics of induced blood-stage malaria studies considered and summary of findings for any participant with grade ≥ 2 levels of alanine or aspartate aminotransferase

Study name	Clinical trial ID*	Test compound	Dose (mg)	*N*	Inoculum (parasites/mL blood)	Treatment day (inoculum D0)	Participants with increased ALT and/or AST (grade ≥ 2): participant, grade, day of peak value (peak value)		ALT:AST ratio
							ALT	AST	
QP13C05	ACTRN12613000565741	Piperaquine	960 (*n* = 5), 640 (*n* = 7), and 480 (*n* = 12)	24	1,800	D7 or D8	R019, grade 2, day 18 (3.7 × ULN)	R019, grade 1, day 18 (2.3 × ULN)	1.6
QP13C14	ACTRN12613001040752	Ferroquine	800	8	1,800	D8	R101(A), grade 4, day 28 (11.6 × ULN)	R101(A), grade 3, day 28 (5.9 × ULN)	1.7
							R104, grade 4, day 12 (13.4 × ULN)	R104, grade 4, day 12 (17.3 × ULN)	0.8
							R105, grade 4, day 13 (11.2 × ULN)	R105, grade 3, day 13 (6.3 × ULN)	1.8
QP14C02	NCT02223871	ACT-451840	500	8	1,800	D7	007, grade 2, day 15 (3.3 × ULN)	007, grade 1, day 10 (1.5 × ULN)	2.1
QP14C11	NCT02281344	MMV390048	20	6	1,800	D7	None	None	–
QP14C12	NCT02389348	OZ439 + DSM265	200+100 (*n* = 8) and 200+50 (*n* = 5)	13	1,800	D7	R201, grade 3, day 23 (5.3 × ULN)	R201, grade 2, day 23 (3.1 × ULN)	1.7
							R204, grade 2, day 12 (3.7 × ULN)	R204, grade 2, day 12 (3.9 × ULN)	0.8
							R103, grade 2, day 28 (3.6 × ULN)	R103, grade 4, day 28 (10.9 × ULN)	0.3
QP14C21	NCT02431637	Piperaquine	480	6	2,800	D7	None	None	–
QP15C01	NCT02543086	Cipargamin + piperaquine	10 on D8 960 on D11–15	8	1,800	D8	5108, grade 4, day 20 (24.1 × ULN)	5108, grade 4, day 20 (11.1 × ULN)	2.2
							5101, grade 3, day 15 (5.2 × ULN)	5101, grade 1, day 15 (2.2 × ULN)	2.4
							5103, grade 2, day 16 (4.7 × ULN)	5103, grade 2, day 16 (2.7 × ULN)	1.8
QP15C05	NCT02431650	Piperaquine + OZ439 or primaquine†	480	11	2,800	D7	R110, grade 3, day 15 (9.3 × ULN)	R110, grade 3, day 15 (5.7 × ULN)	1.6
							R109, grade 2, day 17 (4.2 × ULN)	R109, grade 1, day 10 (2.3 × ULN)	1.8
QP15C20	NCT02867059	SJ733	150 (*n* = 7) and 600 (*n* = 10)‡	15	2,800, Cohort 1	D8	R101(G), grade 4, day 15 (14.6 × ULN)	R101(G), grade 3, day 15 (8.3 × ULN)	1.8
					2,300, Cohort 2		R102, grade 3, day 16 (6.3 × ULN)	R102, grade 2, day 16 (3.0 × ULN)	2.1
							R107, grade 2, day 20 (4.2 × ULN)	R107, normal (1.0 × ULN)	4.0
							R208, grade 2, day 10 (3.0 × ULN)	R208, grade 1, day 10 (1.6 × ULN)	1.9
QP16C04	NCT02783833	MMV390048	40 (*n* = 7) and 80 (*n* = 8)	15	2,800	D8	R502, grade 4, day 13 (13.0 × ULN)	R502, grade 3, day 13 (8.0 × ULN)	1.6
							R503, grade 2, day 10 (4.4 × ULN)	R503, grade 2, day 9 (2.9 × ULN)	1.5
							R505, grade 2, day 17 (2.9 × ULN)	R505, normal (1.2 × ULN)	2.4

ALT = alanine aminotransferase; AST = aspartate aminotransferase; ULN = upper limit of normal. The WHO severity grading was used for ALT and AST: grade 1, 1.25–2.5 × ULN (mild); grade 2, 2.5–5 × ULN (moderate); grade 3, 5.1–10 × ULN (severe); and grade 4, > 10 × ULN (very severe).

* The trials described in this study are registered at ClinicalTrials.gov, with identifiers beginning with NCT or at http://www.anzctr.org.au with identifiers beginning with ACTRN. Cipargamin was previously known as KAE609.

† Participant R110 did not receive primaquine or OZ439, only piperaquine; R109 received piperaquine and then primaquine.

‡ Two participants did not receive SJ733 and were not included in the analysis.

All studies were conducted in accordance with the Declaration of Helsinki and the International Committee of Harmonization Good Clinical Practice Guidelines and received ethical approval from the Queensland Institute of Medical Research Human Research Ethics Committee and the ethical review boards of the study sponsors. All participants provided written informed consent.

### Procedures.

**Figure 1.**
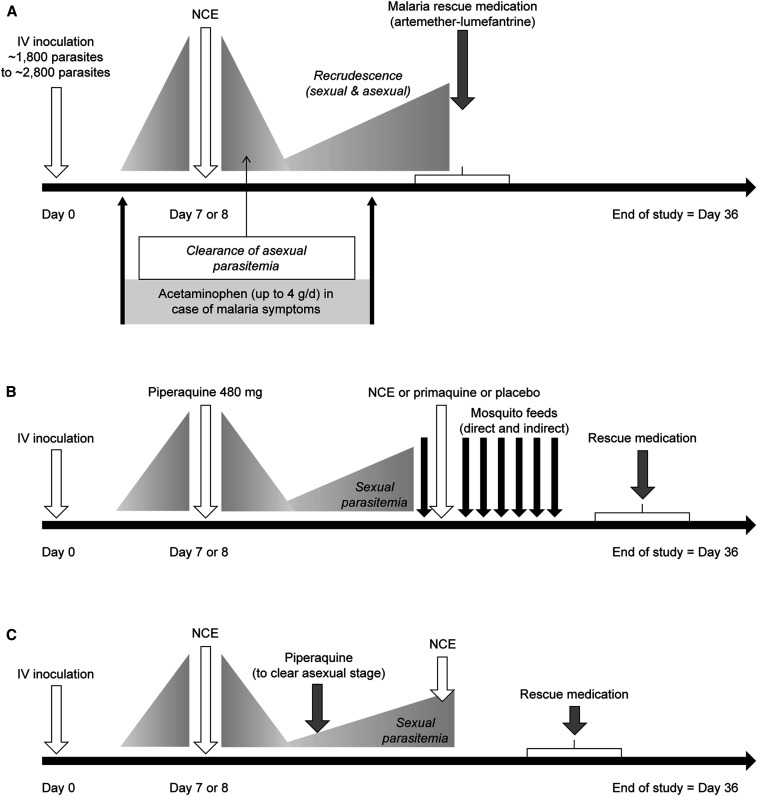
General method for induced blood-stage malaria studies. (**A**) Malaria infection is induced with an intravenous (IV) inoculum of parasitized erythrocytes. After 7–8 days, experimental treatment is given, and if recrudescence occurs, rescue therapy is given. (**B**) The system can be adapted to test for anti-gametocyte activity by administration of piperaquine before the new chemical entity (NCE) to enrich for gametocytes and remove blood-stage parasites; transmission blocking activity can also be tested via membrane feeding assays. (**C**) A combined experiment tests the NCE against blood-stage parasites at a subtherapeutic dose, then primaquine is given at recrudescence, followed by the NCE to test for anti-gametocyte activity.

The general IBSM model is shown in Figure 1, with variations in the initial parasite inoculum and drug treatment day (Table 1).^4^ Adaptions were made to evaluate anti-gametocyte and transmission-blocking activity (Figure 1).^5^ The parasite inoculum was prepared as previously described,^6^ with between ∼1,800 and 2,800 viable intraerythrocytic parasites (*P. falciparum* 3D7) administered to each participant on day 0.^4^ Following establishment of infection, test drugs were administered on day 7 or 8. Parasitemia was determined using real-time quantitative polymerase chain reaction (PCR), as previously described.^7^ Artemether–lumefantrine (Riamet^®^, Novartis) was administered for recrudescence and as rescue therapy at the study end (Figure 1). Frequent blood samples were collected for drug pharmacokinetics and safety biochemistry and hematology laboratory assessments. Participants were confined and monitored for at least 36 hours post-drug administration with ambulatory follow-up until at least day 36.

Retrospective microRNA-122 (miR-122) testing—a safety biomarker for liver cell damage^8^—was performed for all participants in Study QP15C01 (ClinicalTrials.gov NCT02543086). Briefly, RNA was extracted from plasma using the QiAzol extraction method (Qiagen, Valencia, CA), and quantitative PCR analysis was conducted in accordance with the manufacturers’ protocols (Biomark HD System, Fluidigm, San Francisco, CA). For cDNA synthesis of plasma, the input was 3 µL of the RNA preparations in a 5-µL reaction using Megaplex RT Primers Pool A (Life Technologies Corporation, Pleasanton, CA), with addition of a reverse transcriptase primer for mmu-miR-293 for normalization of miR-122 values. This method was validated with a limit of detection threshold value (Ct) for miR-122 of 23 Ct.

### Data collection.

Individual participant demographic data and parasitemia and liver enzyme test results were assembled from study statistical outputs. Baseline values for liver enzyme tests and total bilirubin were recorded on days −2 to 0, before parasite inoculation. Parasitemia levels were determined on day 7 or 8 following inoculation, before treatment with NCE or piperaquine. Peak liver function tests, peak bilirubin, and peak parasitemia were defined as the highest value occurring after day 0. Liver enzyme and bilirubin results were converted to values relative to the upper limit of normal (ULN) (Supplemental Table 1).

### Data analysis and statistics.

The WHO toxicity grading system was used to classify abnormal ALT, AST, and total bilirubin values relative to the ULN: mild (grade 1) 1.26–2.5 × ULN; moderate (grade 2) 2.6–5.0 × ULN; severe (grade 3) 5.1–10 × ULN; and very severe (grade 4) > 10 × ULN.^9^ Values > 2.5 × ULN were considered potentially clinically important. Data analysis used individual participant data and descriptive statistics (MS Excel 2013, Microsoft Corp., Redmond, WA). A retrospective analysis was performed on the effect of acetaminophen on peak ALT/AST levels relative to the ULN using analysis of variance with multiple comparisons test. All enrolled participants who had parasitemia and were treated with an NCE and/or piperaquine were included in the analysis.

## RESULTS

### Participants.

Baseline data for studies included in the QIMRB data review are shown in Table 2. Most participants were males (78.4%; 91/116) and Caucasian (70.0%; 80/116). Two participants in Study QP15C20 did not receive drug treatment and were excluded. Thus, the analysis population comprised 114 participants.

**Table 2:** Participant baseline demographic characteristics

Characteristic	Study name	QP13C05	QP13C14	QP14C02	QP14C11	QP14C12	QP14C21	QP15C01	QP15C05	QP15C20*	QP16C04
	Study drug	Piperaquine (*N* = 24)	Ferroquine (*N* = 8)	ACT-451840 (*N* = 8)	MMV390048 (*N* = 6)	OZ439 + DSM265 (*N* = 13)	Piperaquine (*N* = 6)	Cipargamin + piperaquine (*N* = 8)	Piperaquine + OZ439 or primaquine (*N* = 11)	SJ733 (*N* = 17)	MMV390048 (*N* = 15)
Gender, *n* (%)											
Male		15 (62.5)	3 (37.5)	8 (100)	6 (100)	8 (61.5)	2 (33.3)	8 (100)	11 (100)	17 (100)	15 (100)
Female		9 (37.5)	5 (62.5)	0	0	5 (38.5)	4 (66.7)	0	0	0	0
Mean age (SD) (years)		22.9 (3.5)	26.0 (6.4)	24.1 (6.4)	25.3 (4.0)	26.1 (9.8)	24.3 (4.7)	27.9 (11.8)	28.2 (9.8)	26.6 (9.1)	30.8 (9.0)
Mean body weight (SD) (kg)		68.1 (8.8)	70.6 (9.1)	77.2 (12.0)	81.4 (16.6)	72.0 (11.3)	67.6 (19.8)	79.4 (8.5)	79.9 (12.8)	80.7 (12.8)	79.1 (14.3)
Mean height (SD) (cm)		172.7 (7.7)	173.6 (6.6)	178.8 (9.0)	182.3 (7.5)	173.0 (8.7)	173.3 (12.9)	177.8 (3.9)	178.5 (9.0)	179.4 (6.5)	182.4 (6.6)
Mean BMI (SD) (kg/m^2^)		22.9 (2.5)	23.3 (1.7)	24.1 (2.9)	24.5 (1.8)	23.6 (2.6)	22.0 (2.9)	25.1 (2.7)	25.0 (3.4)	25.1 (3.8)	23.7 (3.8)
Race, *n* (%)											
White		20 (83.3)	7 (87.5)	4 (50.0)	6 (100)	12 (92.3)	6 (100)	4 (50.0)	8 (72.7)	1 (5.9)	12 (80.0)
Asian		1 (4.2)	1 (12.5)	1 (12.5)	0	0	0	0	0	15 (88.2)	0
Other		3 (12.5)	0	3 (37.5)	0	1 (7.7)	0	4 (50.0)	3 (27.3)	1 (5.9)	3 (20.0)

BMI = body mass index.

* Two participants did not receive the new chemical entity dose and were not included in the analysis.

### Liver enzyme elevations in the IBSM model.

**Figure 2.**
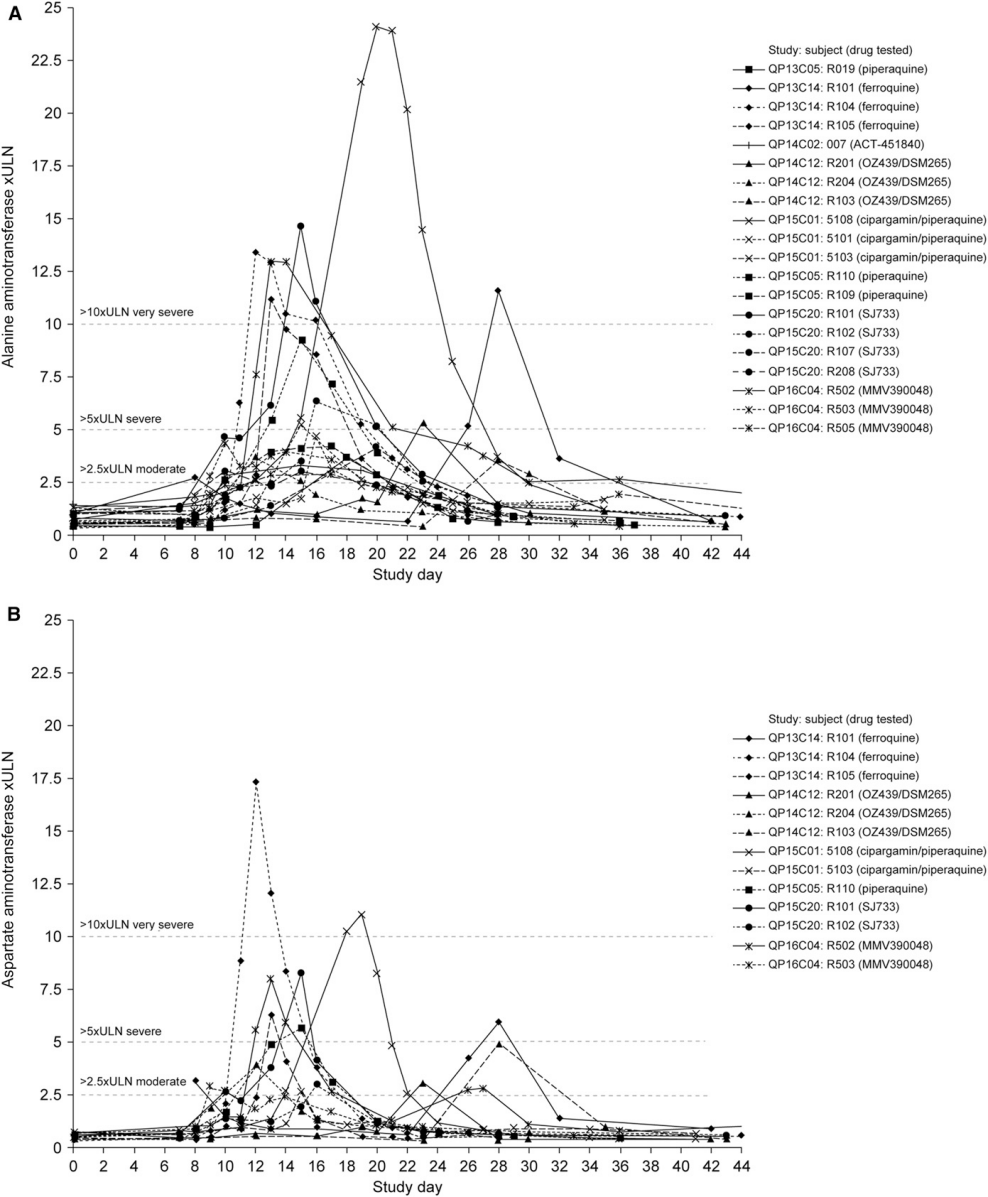
Overview of the time course of changes in liver transaminases for individual participants with moderate or severe increases (≥ 2.6 × ULN; WHO classification) in induced blood-stage malaria studies. (**A**) Alanine aminotransferase (*N* = 20). (**B**) Aspartate aminotransferase (*N* = 13). Malaria inoculum was given on day 0 and study drug administered on day 7 or 8.

Alanine aminotransferase was elevated (> 2.5 × ULN) in 17.5% (20/114) of participants across in eight studies, including seven different NCEs ± piperaquine and piperaquine alone; AST elevations occurred in 11.4% (13/114) of participants, across six studies (Tables 1 and 3). The ALT:AST ratio was > 1 in 17/20 participants with ALT elevations; participants R104 (QP13C14), R103 (QP14C12), and R204 (QP14C12) had ALT:AST ratios < 1 (Table 1). There were no cases where AST was elevated without ALT elevation, whereas seven participants had ALT elevations without increased AST (Table 1, Figure 2).

**Table 3:** Frequency of liver enzyme elevations in healthy participants treated with candidate antimalarial drugs or piperaquine in the induced blood-stage malaria model

Severity	ALT (*N* = 114)	AST (*N* = 114)
Normal	73 (64.0)	93 (81.6)
Grade 1, mild (1.25–2.5 × ULN), *n* (%)	21 (18.4)	8 (7.0)
Grade 2, moderate (2.6–5 × ULN), *n* (%)	10 (8.8)	5 (4.4)
Grade 3, severe (5.1–10 × ULN), *n* (%)	4 (3.5)	5 (4.4)
Grade 4, very severe (> 10 × ULN), *n* (%)	6 (5.3)	3 (2.6)

ALT = alanine aminotransferase; AST = aspartate aminotransferase; ULN = upper limit of normal. Grades are based on the WHO Adverse Event Grading System^9^

In general, ALT started to increase on days 8–10 after inoculation (2–4 days after drug treatment), peaked at days 11–16, declining to normal levels by the study end (Figure 2A). Except for three participants with diverse profiles (R101(A), 5108, and R201), who each received different NCEs, the pattern of ALT elevations showed similar profiles. This suggests that the cause of ALT elevations was not drug specific. Aspartate aminotransferase elevations occurred mainly between days 10 and 16, although were delayed moderately for one participant and more obviously for four participants (Figure 2B).

In participant R103 (Study QP14C12 [OZ439/DSM265]), peak AST was 3-fold higher than ALT, coinciding with elevations in lactate dehydrogenase ([LDH] 4.8 × ULN) and creatine kinase (170.2 × ULN). The participant commenced a weightlifting program 7 days before the biochemical abnormalities, after previously being inactive, and investigators attributed the findings to exercise.

### Potential for severe DILI (Hy’s law).

**Figure 3.**
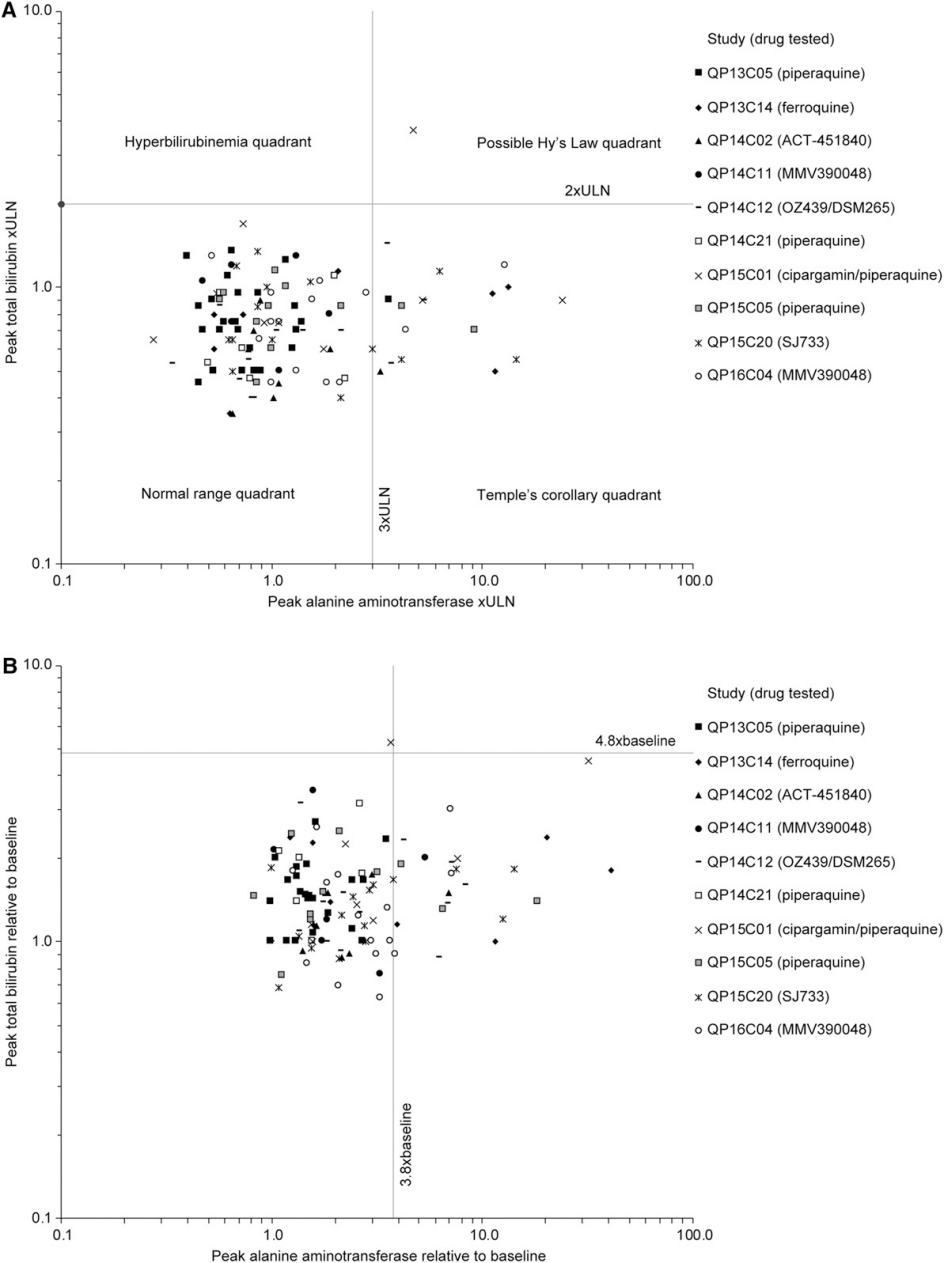
Peak alanine aminotransferase values relative to peak bilirubin levels for induced blood-stage malaria studies. (**A**) Absolute values normalized to the upper limit of normal (ULN). (**B**) Values relative to the baseline.

To investigate the potential for severe liver injury as defined by Hy’s law,^3^ an evaluation of drug-induced serious hepatotoxicity (E-DISH) plot of ALT versus bilirubin relative to the ULN and a modified E-DISH plot (M-DISH) of changes in ALT and bilirubin relative to baseline was performed (Figure 3). Across the IBSM studies, there was one potential Hy’s law case (participant 5103; Study QP15C01; cipargamin–piperaquine) (Figure 3). This 52-year-old man had an undisclosed history of alcohol abuse, and a fatty liver and gallstones observed on ultrasound examination, performed after the ALT/AST elevations were identified. Baseline values (day 8) were mildly elevated for total and conjugated bilirubin (1.4 × ULN and 1.3 × ULN), ALT (1.1 × ULN), and gamma glutamyl transferase (γGT) (1.5 × ULN) (Supplemental Figure 1). Peak elevations in ALT/AST were not observed until 9 days after cipargamin administration and 1 day after piperaquine: AST 2.6 × ULN, ALT 4.7 × ULN, and bilirubin 3.7 × ULN. The participant received two 1-g acetaminophen doses before peak ALT/AST. Total bilirubin mostly comprised unconjugated bilirubin. This was not suggestive of global liver function loss and inconsistent with DILI, being more likely explained by hemolysis or impaired bilirubin conjugation, as seen with Gilbert’s syndrome. Subsequently, plasma miR-122 levels were assessed in all eight cohort participants; correlation between miR-122 and ALT profiles may reflect hepatocyte leakage or active release as a major cause for ALT elevations.^8^ In this case, the miR-122 profile did not match ALT, suggesting that bilirubin elevation was probably not caused by hepatocyte injury (Supplemental Figure 2). It was not possible to determine the mechanism underlying the liver function test abnormalities. Drug-induced liver injury expert review did not consider this a Hy’s law case because of the bilirubin profile and history of alcohol abuse, and although a contribution for cipargamin–piperaquine could not be excluded, the cause was likely multifactorial.

### Parasitemia.

**Figure 4.**
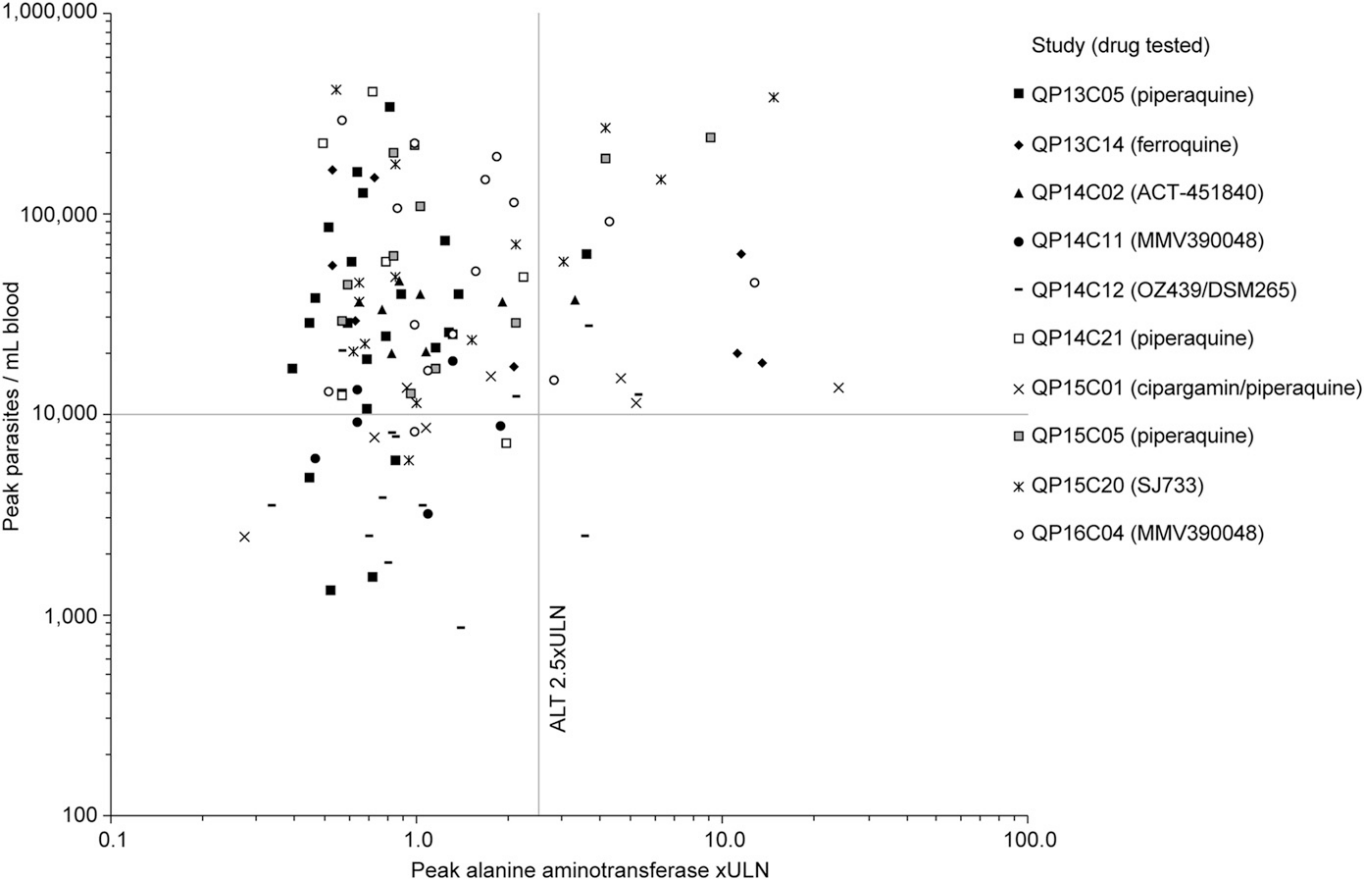
Relationship between peak parasitemia and alanine aminotransferase values in induced blood-stage malaria studies.

Across the IBSM studies, 19/20 cases of moderate-to-severe ALT elevation occurred when participants had peak parasitemia levels greater than 10,000 parasites/mL (Figure 4). The relative risk of parasitemia > 10,000 parasites/mL for causing moderate/severe ALT increases was 4.8 (0.68–34.0, *P* = 0.116) (Table 4). Parasite-induced hemolysis was also considered as a possible cause of ALT/AST elevations. Hemolysis index was increased in 29/114 participants, although the proportion of participants with an elevated hemolysis index was similar in those with moderate-to-severe ALT elevations (30.0% [6/20]) versus those with mild elevations/normal ALT (24.5% [23/94]).

**Table 4:** Relative risk of increased alanine aminotransferase with different cofounding factors in induced blood-stage malaria models

Risk factors	Grade 2, 3, or 4 ALT (*n* = 20)	Normal or grade 1 ALT (*n* = 94)	Relative risk (95% CI)	*P*-value
Parasitemia ≥ 10,000 parasites/mL	19	72	4.8 (0.68 to 34.0)	0.116
Acetaminophen > 6 g	4	6	2.6 (1.08 to 6.29)	0.034
Parasitemia ≥ 10,000 parasites/mL plus acetaminophen > 6 g	4	5	2.9 (1.24 to 6.88)	0.015

ALT = alanine aminotransferase.

Further analysis was conducted on Study QP15C20 (SJ733), as different parasite inoculum levels were used in this study. Participants in Cohort 1 (*n* = 7) received a single 150 mg SJ733 dose on day 8, following inoculation with 2,800 *P. falciparum* on day 0. Three participants from Cohort 1 (R101, R102, and R107) showed moderate-to-severe ALT/AST elevations, but no change in bilirubin, with relatively high-peak parasitemia levels (147,074–378,077 parasites/mL). As participants in Cohort 2 (*n* = 8) would receive a higher SJ733 dose (600 mg on day 8), they were inoculated with only 2,300 parasites. In Cohort 2, peak parasitemia levels were between 5,862 and 69,947 parasites/mL, with one participant (R208) having a moderate ALT elevation (3.0 × ULN). The greater antimalarial effects of the higher SJ733 dose in Cohort 2 resulted in more rapid parasite clearance versus Cohort 1. In summary, compared with Cohort 1 where three participants had AST/ALT elevations, in Cohort 2, a 4-fold higher dose with a greater antimalarial effect and a lower parasite inoculum size and peak parasitemia did not induce severe ALT/AST elevations.

**Figure 5.**
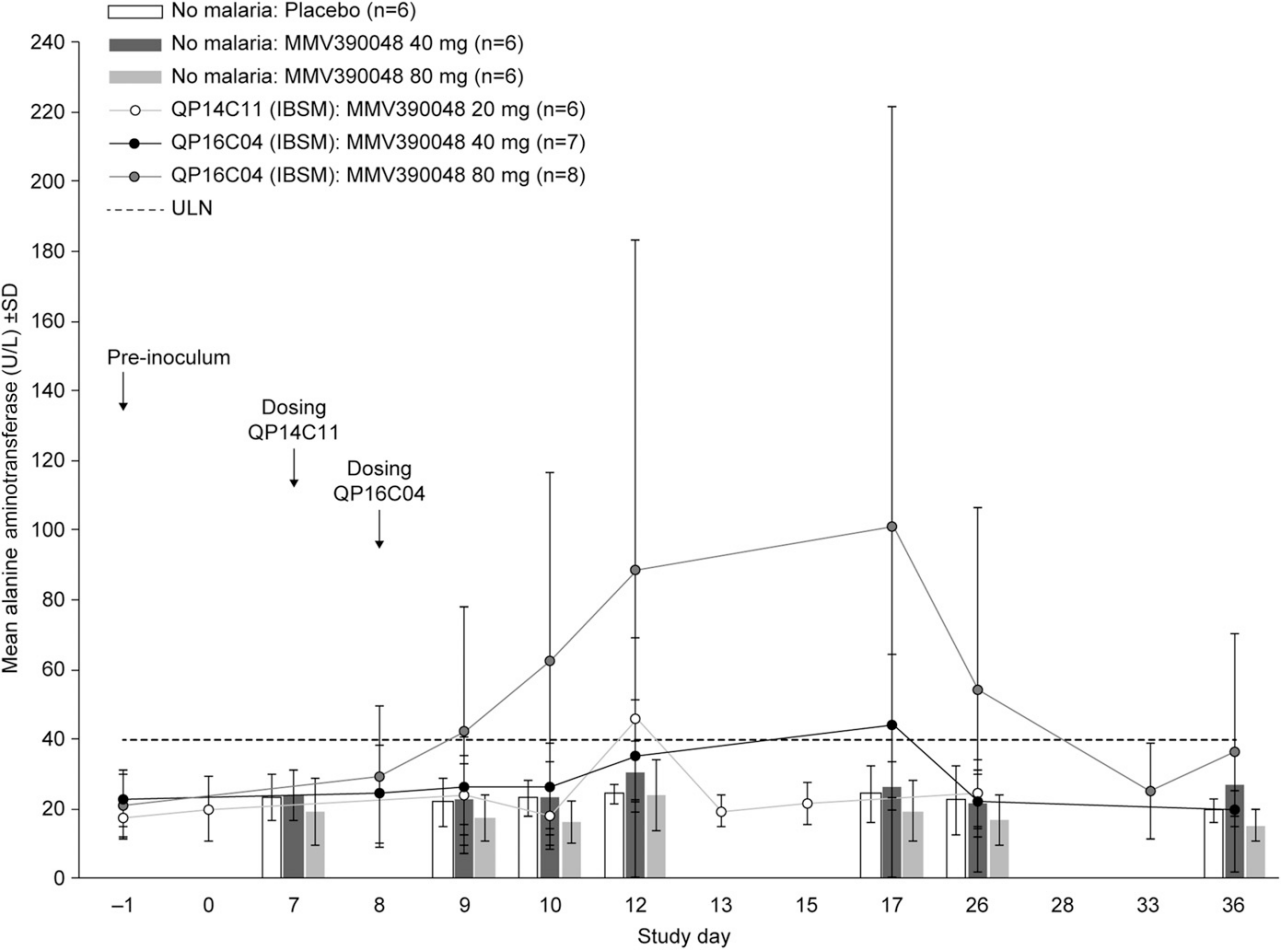
Mean alanine aminotransferase values following a single oral dose of MMV390048 (40 mg or 80 mg) given to participants with previous malaria inoculation (lines) or without malaria inoculation (bars). Inoculum intravenous administration was performed on Day 0.

A similar analysis was conducted for studies with the NCE MMV390048 (QP14C11 and QP16C04). In Study QP14C11 (*n* = 6), a 1,800 parasite inoculum with treatment on day 7 and a 20 mg dose, caused a mild ALT increase in one participant (1.1 × ULN). In Study QP16C04, MMV390048 40 mg (Cohort B1) or 80 mg (Cohort B2) was administered on day 8, with a 2,800 parasite inoculum. In Cohort B1 (*n* = 7), there were no ALT/AST elevations of grade 2 or higher. In Cohort B2 (*n* = 8), three participants showed ALT elevations; one severe (R502: 13.0 × ULN; WHO grade 4) and two moderate (grade 2, R503: 4.4 × ULN; R505: 2.9 × ULN). This might appear to be a simple dose–response, with ALT/AST elevations more frequent at the higher MMV390048 dose. However, the same doses (40 and 80 mg) were tested in a Phase I study in healthy participants (ClinicalTrials.gov identifier: NCT02230579). In this case, there was no increase in ALT, with values similar to those obtained with placebo (Figure 5). This suggests that ALT increases were not a simple dose–response effect but related to the combination of malaria and drug.

### Acetaminophen administration.

**Figure 6.**
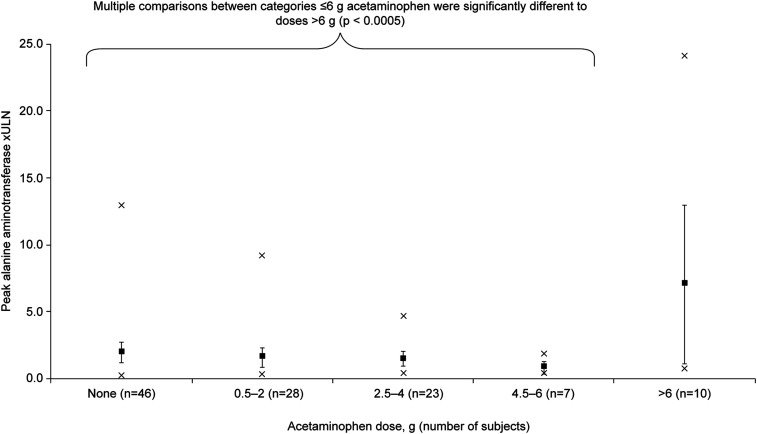
Relationship between cumulative acetaminophen dose and peak alanine aminotransferase levels relative to the upper limit of normal. Mean values ± 95% CI; crosses are the maximum and minimum values.

Most participants (59.6%; 68/114) in the IBSM studies received acetaminophen for malaria symptoms. Administration started with symptom onset around day 6, before test drug administration, and continued as required. In most cases, acetaminophen was given before ALT/AST increases were observed. In the six studies reporting severe ALT increases (≥ 5.1 × ULN), 6/10 participants received acetaminophen at cumulative doses of 2 to 8.5 g (Supplemental Table 2). There was a statistically significant increase in peak ALT values relative to the ULN with cumulative acetaminophen doses > 6 g (mean 7.0 × ULN [95% 1.1, 12.9]) versus 0 to ≤ 6 g (mean 1.7 × ULN [95%CI 1.3, 2.1]) (*P* < 0.0005) (Figure 6). Adjustment for cohort and gender did not alter these results (data not shown). This translated into a significantly increased relative risk of ALT elevations with acetaminophen doses > 6 g versus lower doses or no acetaminophen (Table 4). Notably, relative risk further increased among participants with acetaminophen doses > 6 g and parasitemia > 10,000 parasites/mL blood (Table 4).

Following the initial cases of ALT/AST elevations in studies QP13C14 and QP15C01, further investigation of samples from Study QP15C01 (cipargamin) showed an apparent temporal correlation between acetaminophen dosing and acetaminophen adduct levels (Supplemental Figure 3). However, the observed acetaminophen adduct levels (≤ 0.1 µmol/L) were not in the range associated with clinically relevant liver toxicity.

### Piperaquine.

Piperaquine data were examined because it has no known DILI risk, despite extensive use in acute uncomplicated malaria.^10^ In Study QP15C01, NCE (cipargamin) was given on day 8 at subtherapeutic doses for pharmacokinetic/pharmacodynamic evaluation. Piperaquine 480 mg was administered 3–7 days after cipargamin to clear asexual parasites, while permitting gametocyte emergence. Following intentional evaluation of low-dose (10 mg) primaquine in the initial cohort to observe recrudescence, a repeat therapeutic dose of cipargamin was planned to test anti-gametocyte activity in the same participants. However, this repeat cipargamin dose was not given, as increases in ALT/AST were observed in 3/8 participants in Cohort 1 (5101, 5103, and 5108), requiring early study termination. The predominant effect was on ALT, but AST was also elevated, with levels rising after PCR-documented recrudescence and shortly after piperaquine administration. Participant 5103 had additional risk factors for ALT/AST increases, as discussed earlier, but in all three participants, the elevation in liver transaminases accelerated following piperaquine administration. Participants 5101 and 5108 had matching miR-122 and ALT profiles (Supplemental Figure 2), and hepatocyte leakage/release was assumed as the underlying reason for ALT elevations; reduced ALT clearance could not be the only cause. The other five participants in the cohort, who had no liver signals, had normal miR-122 levels. The ALT/AST increases following piperaquine appeared to be associated with parasitemia reduction by an effective antimalarial drug.

Three additional piperaquine studies were examined. In QP15C05, 2/12 participants showed ALT/AST elevations (R109: ALT 4.2 × ULN, R110: 9.2 × ULN) after 480 mg piperaquine administration on day 8 and a 2,800-parasite inoculum. QP13C05 had a lower inoculum size (1,800 parasites), with piperaquine 480–960 mg administered on day 7 or 8, with one instance of moderately increased ALT (R019: ALT 3.7 × ULN). In QP14C21, a piperaquine dose of 480 mg on day 7 and an inoculum of 2,800 parasites produced no ALT/AST elevations. Thus, earlier treatment (day 7) and lower inoculum size (1,800 parasites) appeared to be protective against piperaquine induced ALT/AST elevations.

## ANALYSIS OF POTENTIAL CAUSES OF LIVER ENZYME ELEVATIONS IN THE IBSM MODEL

### Participant susceptibility.

Across the studies examined, only one participant (5103; Study QP15C01 [cipargamin–piperaquine]) had elevated ALT and bilirubin (Figure 3), considered by DILI Experts to be caused by undisclosed alcohol abuse. One participant (R103; Study QP14C12 [OZ439/DSM265]) had an AST increase co-incident with exercise, with typical increases in LDH and creatine kinase. Strenuous exercise is a known cause of liver enzyme increases, commonly observed in Phase I studies.^11^

The typical IBSM study protocol already excludes participants with known increased hepatotoxicity risk. However, additional participant-specific factors could predispose to liver injury during antimalarial drug treatment. These factors could be genetic,^12^ such as DILI caused by antituberculosis drugs in patients with the slow acetylator phenotype resulting from NAT2 variants,^13,14^ nonalcoholic steatohepatitis (NASH),^15,16^ or other conditions that would lead to exclusion but remain unknown or undisclosed by the participant. Age and gender may also play a role in DILI risk.^17,18^ However, apart from the two cases discussed, such participant-specific factors are an unlikely explanation for the high proportion of participants exhibiting increased ALT/AST in the IBSM studies described.

### Presence of malaria.

In Study QP15C20 (SJ733), a lower drug dose and higher inoculum size induced severe ALT/AST elevations, whereas a higher dose with a lower inoculum size did not. Similarly, in studies QP14C11 and QP16C04 (MMV390048), and in Study QP15C01 (cipargamin–piperaquine), the same doses that had no effect on ALT/AST levels in healthy participants without malaria caused ALT/AST elevations in healthy participants who were inoculated with malaria. Although there was a trend for ALT/AST elevations in participants with parasitemia levels > 10,000 parasites/mL, most participants (76.6%; 72/94) without ALT elevations also had parasitemia levels above this level. Thus, although there may be some relationship between elevated liver enzymes and malaria in the IBSM data, it is not simply related to parasitemia level. Mild hemolysis occurred in some participants and dysregulation of iron detoxification is a possible cause of increased ALT/AST.^19^ Although there was no relationship between ALT/AST increases and hemolysis index, we cannot determine whether some participants were particularly sensitive to the effects of hemolysis, and so a relationship between hemolysis and ALT/AST increases cannot be excluded.

Adults and children living in endemic areas with acute uncomplicated *P. falciparum* malaria commonly experience mild ALT/AST elevations (up to 2.5 × ULN; WHO Grading), and severe falciparum malaria is a known risk factor for liver injury.^20,21^ In the IBSM model, parasitemia in nonimmune participants is much lower at the time of test drug administration (mostly < 100,000, rarely up to 400,000 parasites/mL) than observed for uncomplicated malaria in semi-immune patients from endemic areas (typically 1,000,000–100,000,000 parasites/mL). However, drug-induced parasite clearance kinetics in the IBSM model closely mimic those reported in malaria patients.^1^ Notably, direct inoculation of infected erythrocytes in the IBSM model eliminates the prepatent period for parasitemia development (i.e., liver stage of the disease). Most IBSM study participants experience some inflammation-related clinical symptoms, including malaise, chills, fever, headache, neck ache, myalgia, and back ache from day 7 onward.^4^ Thus, inflammation is evident after intravenous inoculation (day 0) at the point when drug treatment is initiated (day 7/8).

A possible explanation for transient ALT/AST elevations without impact on bilirubin in the IBSM model is that malaria inoculation induces acute inflammation and oxidative stress in healthy nonimmune participants.^19^ It is also possible that this inflammatory state could increase participant susceptibility to drug-induced effects on the liver from acetaminophen or test drugs or that there is some unappreciated individual predisposition to this phenomenon.

To better understand the impact of malaria on ALT/AST and bilirubin in nonimmune participants, a retrospective analysis was performed of hospitalized travelers with acute malaria returning from endemic areas to Queensland, Australia, between 2006 and 2016.^22^ For this largely malaria-naïve population, ALT and/or AST elevations ≥ 3.1 × ULN occurred in 15.1% (*n* = 130/861) and 14.8% (127/861) of patients, respectively, with an incidence reported to day 7 following diagnosis.^22^ When stratifying by severity, ALT elevations were ≥ 3.1 to ≤ 5.0 × ULN in 8.8% (76/861) of patients, ≥ 5.1 to ≤ 10 × ULN in 4.7% (41/861), and > 10 × ULN in 1.5% (13/861). The highest peak ALT elevation identified was 25.8 × ULN on day 4. Of the 130 cases with elevated ALT, 64.6% (84/130) peaked between days 0 and 3 and 35.4% (46/130) between days 4 and 11; all largely resolved by day 28.^22^ Note that these timings are synchronized with diagnosis, which occurred only after symptom onset, so cannot be directly compared with the current analysis where day 0 represents parasite inoculation.

Similarly, a recent review of 217 patients with imported uncomplicated *P. falciparum* malaria presenting at hospitals in the Netherlands between 2002 and 2017, showed moderate increases in liver function tests (ALT/AST/γGT/ALP) at admission in 14.0% (26/186), and severe increases in 4.8% (9/186).^23^ These increases were likely related to malaria hepatopathy. Notably, there was a positive association between parasite density on admission day and liver function test abnormalities (*r* = 0.25, *P* = 0.005).^23^

Thus, it appears that up to 15% of malaria-naïve patients with imported uncomplicated *P. falciparum* malaria have ALT/AST increases, which may be related to parasitemia levels. These findings are consistent with the present analysis of participants in the IBSM model, that is, ALT increased in 17.5% (20/114) and AST increased in 11.4% (13/114), with a trend for these to occur at higher parasite densities.

### Acetaminophen administration.

Acetaminophen has a known risk of dose-related intrinsic DILI, caused by acetaminophen–protein adduct formation.^24^ Acetaminophen causes pronounced ALT elevations in healthy participants with sub-chronic daily administration (4 g/day for > 4 days), in a time frame consistent with those observed in the IBSM studies.^25,26^ In rodents, an induced inflammatory state lowers the toxicity threshold for several direct hepatotoxins, including acetaminophen.^27–29^ It is, therefore, possible that malaria-associated inflammation could exacerbate this drug-induced effect. Alternatively, acetaminophen administration could be regarded as an indirect measure of inflammation and immune activation, higher doses likely being given to participants who had more malaria symptoms. None of the IBSM studies evaluated inflammatory markers, so this cannot be directly evaluated. It is also possible that acetaminophen increased sensitivity to NCE toxicity through additive depletion of glutathione by both drugs. Thus, acetaminophen could be considered as a risk factor for ALT elevation independently or in combination with malaria inoculation and/or test drugs.

### Study drug.

The ALT/AST elevations observed with piperaquine were unexpected as dihydroartemisinin–piperaquine is used to treat uncomplicated malaria without any evidence of a liver safety signal. This suggests that hepatotoxicity is not inevitably related to the study drugs. Also, it indicates that liver safety findings in IBSM studies do not necessarily translate into hepatotoxic risk in malaria patients. However, for the NCEs tested, their possible contribution to the observed liver toxicity cannot be excluded because of the absence of a control group.

Transient ALT/AST abnormalities are reported following antimalarial therapy of acute uncomplicated malaria and are severe in between 0.2 and 0.7% of patients.^30^ These changes are not associated with DILI and are not more common on repeated drug treatment.^30,31^ Severe ALT/AST increases following antimalarial therapy appear to occur more frequently in nonimmune versus semi-immune malaria patients^22^ and in children versus adults in malaria endemic regions.^31^ Whether there is a common underlying disease-related mechanism across antimalarial drug therapies which drives these ALT/AST abnormalities and/or whether they result from direct drug-specific hepatotoxicity is unclear. These observations suggest that pharmacological treatment of acute uncomplicated malaria in itself is associated with additional stress for hepatocytes.

## DISCUSSION OF PRIOR EVIDENCE FOR LIVER ENZYME ELEVATIONS IN VOLUNTEER INFECTION STUDIES

To further examine possible explanations for increased liver enzymes in the IBSM studies, we considered previous reports of ALT/AST elevations in VISs. This was not a formal systematic data review but aimed to extend the discussion of the observed liver enzyme elevations in the QIMRB IBSM studies. Relevant articles were identified by consultation with experts and investigators involved in VIS models. Also, a search was conducted of PubMed citations between June 1960 and September 2018 with the terms: challenge, inoculation, IBSM, CHMI, sporozoites, liver, toxicity, hepatotoxicity, malaria, aminotransferase, transaminases, challenge, and *Plasmodium*. Authors were not contacted for data sharing.

### Induced blood-stage malaria studies.

Most IBSM studies have been conducted at QIMRB. However, there are 12 published reports of IBSM studies conducted in the 1970 and 1980s by the United States of America Army.^32–43^ Unfortunately, only limited biochemistry data were available from two of these studies, and the incidence of ALT/AST elevations cannot be estimated.^33,39^

### Sporozoite challenge model—*Anopheles* bite.

Induction of malaria infection in human participants via the bites of infected *Anopheles* mosquitoes was first used to test antimalarial activity by the U.S. Army in the 1970s. It has since been used extensively for evaluating chemoprevention strategies with registered antimalarial drugs or NCEs.

A recent study examined the effect of marketed antimalarial drugs on liver function in healthy malaria-naïve participants aged 18–35 years participating in VISs using the *Pf*SPZ challenge methodology, mainly the *Anopheles* bite model, conducted between 2001 and 2016 at Radboud University, the Netherlands. Liver function tests (ALT/AST/γGT/ALP) were moderately increased in 10.2% (19/187) of participants, with severe (grade 3) increases reported in 8.6% (16/187).^23^ Liver function test abnormalities peaked soon after treatment initiation, regardless of drug regimen, and returned to normal within 3 to 6 weeks. There was a positive association between liver enzyme elevations and parasite burden (*P* < 0.001), as well as cumulative inflammatory cytokine responses (*r* = 0.65, *P* = 0.008), and oxidative stress markers (*r* = −0.63, *P* = 0.001).^23^ These findings are, therefore, consistent with observations from the IBSM studies conducted at QIMRB, Australia.

In addition to the aforementioned review, we identified four reviews/meta-analyses with vaccine studies,^44–47^ a more recent vaccine study,^48^ 19 drug studies,^35–42,49–59^ and six studies intended to validate/standardize methods for the *Pf*SPZ *Anopheles* bite model.^60–65^ There was a surprising lack of data on ALT/AST levels; for the drug studies, 13/19 (68.4%) publications omitted laboratory safety data.

One study with laboratory data included piperaquine. In a randomized trial of four antimalarial drug regimens to show feasibility of enriching for gametocytes, low-dose piperaquine (480 mg) or sulfadoxine–pyrimethamine (500 mg/25 mg) were given, followed by a curative regimen on recrudescence.^59^ In the two low-dose piperaquine-containing regimens, ALT elevations (grade 2/3; WHO) occurred in 37.5% (3/8) of participants and AST elevations in the same proportion. Across all four regimens, ALT/AST elevations occurred in 33.3% (4/12) of participants.^59^ Thus, just as for the IBSM model, piperaquine administration during blood-stage malaria was associated with increases in liver enzymes in the *Pf*SPZ *Anopheles* bite model.

Although placebo controls are often lacking in VISs, one randomized double-blind placebo-controlled chemoprevention study was conducted with pafuramidine.^55^ In this case, ALT/AST levels were elevated in 10/15 (66.6%) healthy participants who developed malaria, of whom 3/5 (60.0%) were in the placebo arm and 7/14 (50.0%) in the pafuramidine arms.^55^ Four participants (1 placebo/3 pafuramidine) had ALT increases of WHO grade 3 severity (5.1–10 × ULN), with maximum increases of approximately 8 × ULN (Supplemental Table 3).^55^ The observation of moderate/severe ALT/AST elevations in placebo-controlled participants suggests a contribution of the study procedures (inoculation and/or acetaminophen).

### Sporozoite challenge model—Systemic administration.

*Plasmodium falciparum* sporozoite challenge by systemic administration uses infectious, aseptic, purified, cryopreserved *P. falciparum* NF54 sporozoites. The number administered varies according to the administration route: 3,200 intravenously and 75,000 by intramuscular injection,^66^ and although the number needed by intradermal administration has not been fully established, it exceeds 25,000.^67–69^ Unlike the IBSM methodology, *Pf*SPZ challenge includes a prepatent liver stage before parasitemia is detected and malaria symptoms emerge. Except for one study of chloroquine prophylaxis,^70^ and two recent studies evaluating chemoprevention with DSM265,^58,71^ antimalarial drug activity has not been tested in this model. Rather, approved antimalarial drugs are administered as rescue therapy immediately once malaria parasites are detected in blood smears or using PCR. Thus, parasitemia levels on the treatment day are usually low; generally < 10,000 and often < 1,000 parasites/mL.

We reviewed 14 publications of interest concerning *Pf*SPZ challenge by systemic administration in nonimmune healthy participants.^58,66–78^ Laboratory safety data were often absent or insufficiently detailed during the timeframe of interest. When adverse events categorized under investigations or laboratory safety data were provided, aminotransferase elevations above the ULN were noted in nonimmune individuals at a rate of 0–61%.^58,66,67,69,71–74,77^ Severity grading information was not often available, although an ALT elevation of 16.6 × ULN was noted in one study with atovaquone–proguanil.^67^ No data were available for acetaminophen dosing for any of the published studies.

## RECOMMENDATIONS

Specific safety provisions for VISs with NCEs are proposed to minimize hepatotoxicity risk in study participants. Based on our analysis and published data, these provisions should be applied to both IBSM and *Pf*SPZ models. Also, recommendations are made to maximize the opportunity to differentiate between potential DILI and model-related factors (Box 1). For drugs with hepatotoxic potential identified in preclinical species, a careful risk:benefit analysis is required. This might conclude that VISs be avoided or necessitate a modified design, for example, sequential enrolment to ascending doses following safety review.

**Box 1 Summary of recommendations**

**Table:** 

#	Recommendation	Contributors
1	Exclusion of high-risk participants	PI and sponsor
2	Enhanced informed consent process	PI and sponsor
3	Control group and comparable data from Phase I studies	PI and sponsor
4	Control parasitemia levels at treatment administration	PI, sponsor, and experts
5	Symptomatic treatment with ibuprofen rather than acetaminophen	PI and sponsor
6	Laboratory tests and biomarkers	PI, sponsor, and experts
7	Presentation of study results to standard format	PI, and sponsor

PI = principal investigator.

### Exclusion of high-risk participants.

As the liver is involved in malaria, every precaution should be undertaken to exclude participants at greater risk of liver injury. Obtaining a minimum of two baseline samples before malaria inoculation at a 48- to 72-hour interval should identify participants with fluctuating ALT/AST exceeding > 1.25 × ULN. Mild baseline ALT/AST elevations can indicate underlying hepatic stress, for example, caused by NASH or alcoholism.^79^ Induced blood-stage malaria study protocols should stipulate that ALT/AST elevations above acceptable values before the inoculum day should not be infected with malaria. After parasite inoculation, pretreatment ALT/AST and bilirubin levels should be obtained and reviewed by the investigator. Alanine aminotransferase/AST may be tested up to 24 hours before planned NCE administration, and if above acceptable limits, standard of care (i.e., a registered antimalarial) can be administered instead of the NCE. If ALT/AST is elevated following inoculation and/or treatment, retesting should be conducted within 24 hours to exclude laboratory errors and reviewed for possible participant-dependent causes.

#### Enhanced informed consent process.

Healthy participants enrolled in VISs are already informed that asymptomatic transient ALT/AST elevations may occur. Enhanced consent should highlight the potential severity of ALT/AST elevations and the consequences of withholding key information. Where an NCE is to be administered, alcohol, drugs of abuse, and more generally other medication, should be strictly avoided during the study and the follow-up period if possible. Alcohol and drugs of abuse screening tests with immediate reading (breath tests and urine dipstick, respectively) should be performed not just at screening, but at key time points, for example, pre-inoculation or pre-dose. Participants with a positive test should be withdrawn from the study, but will need treatment with a standard antimalarial if already inoculated with malaria.

#### Control group and comparable data from Phase I studies.

A control arm, including either placebo or a registered antimalarial, would allow assessment of drug-dependent ALT/AST elevations versus those caused by model conditions. Although a control group increases costs, potential delays in drug development while investigating elevated ALT/AST values are also costly. It may be possible to use an historical control group with a marketed antimalarial or placebo/rescue medication where all other covariates (inoculum size, treatment day, and concomitant medications) are equivalent. It would also be valuable to ensure that VIS design is comparable with the Phase I first-in-human study in terms of dose regimen and patient population. Thus, any liver signals from the VISs can be interpreted relative to similar participants who are not infected with malaria.

#### Control parasitemia levels at treatment administration.

Although a threshold value has yet to be established, limiting parasitemia levels to < 10,000 parasites/mL blood would be precautionary. The feasibility of initiating NCE treatment at a predefined parasitemia threshold should be explored. Further work is needed to understand whether specific inflammatory/immune markers and/or malaria clinical score could be used to predict liver stress and integrated into a treatment decision tree. Parasite clearance kinetics for individual drugs, that is, fast or slow acting, could also influence the hepatic response to parasite clearance, and this requires further investigation.

#### Symptomatic treatment with ibuprofen rather than acetaminophen.

Owing to the known effects of acetaminophen and adducts on hepatocytes, ibuprofen is suggested as an alternative for managing malaria symptoms after inoculation. Treatment with ibuprofen is more effective than acetaminophen in lowering temperatures in malaria patients throughout the first 4.5 hours after dosing.^80^ Ibuprofen can be safely administered to healthy participants in a fasted state without increased risk of gastrointestinal toxicity.^81^ Alanine aminotransferase elevations can occur with ibuprofen doses of 2.4–3.2 g daily, but are generally mild.^82^ However, potential adverse effects, such as the known association of NSAIDs with acute kidney injury,^83^ requires specific evaluation in VISs.

#### Laboratory tests and biomarkers.

A systematic liver safety panel, serum LDH, hemolysis panel, hemolysis index, and haptoglobin, along with parasite density and malaria clinical score should comprise VIS safety endpoints, with an appropriate decision tree if clinically relevant effects are detected. Observation of an ALT elevation > 5 × ULN should prompt measurement of additional liver safety markers for mechanistic evaluation through a decision tree provided in the VIS protocol. For example, novel serum liver safety biomarkers investigated by Innovative Medicines Initiative–Safer and Faster Evidence-based Translation and Critical Path Institute’s Predictive Safety Testing Consortium and endorsed by FDA/EMA could be considered.^84–86^ Biomarkers should be measured in the entire cohort, that is, including participants with and without elevated ALT/AST values, to allow comparison.

#### Presentation of study results to standard format.

Summarized laboratory safety data with standardized severity gradings, along with parasitemia levels, malaria clinical score, and concomitant medications for all VIS participants should be included in preliminary datasets provided to the Safety Review Team, presented in clinical study reports, and published with manuscripts as Supplementary Data. Publications should state the adverse event severity grading system used. For any participants with elevated ALT/AST, all key parameters should be fully documented/reported, including the time course of changes in liver enzymes, parasitemia, medications received, malaria clinical score, and pharmacokinetic/pharmacodynamic data. A standardized set of tables and graphical outputs capturing the key findings and covariates is recommended.^87^ For comparability, data should be reported with reference to the ULN.

## CONCLUSION

The evidence reviewed suggests that IBSM study procedures (i.e., presence of induced malaria and/or acetaminophen administration) may contribute to ALT/AST elevations in the IBSM model. Similar findings are reported from *Pf*SPZ VIS models, both systemic administration and mosquito bite. However, based on the available evidence, the possibility that the test drugs could account for ALT/AST elevations cannot be formally excluded. The recommendations outlined include measures to minimize participant risk and aid the interpretation of findings suggestive of hepatotoxicity. Asymptomatic ALT/AST increases of limited severity should not have a major impact on NCE risk/benefit, or halt drug development. However, any findings suggestive of hepatotoxicity in VISs will need careful evaluation in Phase I trials and in comparison with existing antimalarial drugs in semi-immune malaria patients. In addition, the risk:benefit analysis of potentially hepatotoxic NCEs in VISs requires careful evaluation.

## Supplemental data, tables, and figures

Supplemental materials
