# Supplementary material for: Liver Enzyme Elevations in Plasmodium falciparum Volunteer Infection Studies: Findings and Recommendations
Source: Am J Trop Med Hyg. 2020 Apr 20;103(1):378–93. doi: 10.4269/ajtmh.19-0846 (PMC7356411; doi:10.4269/ajtmh.19-0846)
Supplement: Supplementary file 1 [file tpmd190846.SD1.pdf]

Supplemental material

Liver enzyme elevations in *Plasmodium falciparum* volunteer infection studies: findings and recommendations

Farouk M Chughlay, Samantha Akakpo, Anand Odedra, Katalin Csermak-Renner, Elhadj Djeriou, Cornelis Winnips, Didier Leboulleux, Aditya H. Gaur, G. Dennis Shanks, James McCarthy, Stephan Chalon

Supplemental Table 1. Values for the upper limit of normal for each study included in the analysis.

Supplemental Figure 1. Profile for Subject 5103 in Study QP15C01. A: Changes in laboratory parameters; B: Concomitant medication; C: Adverse events of any cause.

Supplemental Figure 2. Temporal relationship between miR122 and ALT levels in subjects with increased ALT levels in study QP15C01.

Supplemental Table 2. Acetaminophen administration to subjects with moderate (Grade 2) or severe (Grade 3 or 4) increases in ALT in IBSM studies.

Supplemental Figure 3. Temporal relationship between acetaminophen dosing and presence of adducts in study QP15C01.

Cipargamin was administered on Day 8 at a sub-therapeutic dose. Subjects 5101, 5103 and 5108 had moderate-to-severe increases in ALT.

Supplemental Table 3. Data from a study of pafuramidine in the *Anopheles*-bite sporozoite challenge model. A) Risk of increased aminotransferase levels in subjects with or without malaria treated with pafuramidine and/or acetaminophen. B) Severity of changes in liver system laboratory measures with pafuramidine or placebo. Adapted from Nyunt MM, et al. Am J Trop Med Hyg 80:528-535.

Supplemental Table 1. Values for the upper limit of normal for each study included in the analysis.

Study	Test compound	Upper limit of normal males, U/L			Upper limit of normal females, U/L		
		ALT	AST	Bilirubin	ALT	AST	Bilirubin
QP13C05	Piperaquine	40	40	20	30	35	20
QP13C14	Ferroquine	40	40	20	30	35	15
QP14C02	ACT-451840	40	40	20	NA	NA	NA
QP14C11	MMV390048	39.3*	39.3*	20	NA	NA	NA
QP14C12	OZ439 + DSM265	39.3*	39.3*	20	29.5*	34.4†	15
QP14C21	Piperaquine	40	40	20	30	35	15
QP15C01	Cipargamin + piperaquine	40	40	20	NA	NA	NA
QP15C05	Piperaquine	40	40	20	NA	NA	NA
QP15C20	SJ733	40	40	20	NA	NA	NA
QP16C04	MMV390048	40	40	20	NA	NA	NA

NA, not applicable as no females were recruited to this study. *0.668 ukat/L for males, 0.501 ukat/L for females. †0.5845 ukat/L.

Supplemental Figure 1. Profile for Subject 5103 in Study QP15C01. A: Changes in laboratory parameters; B: Concomitant medication; C: Adverse events of any cause.

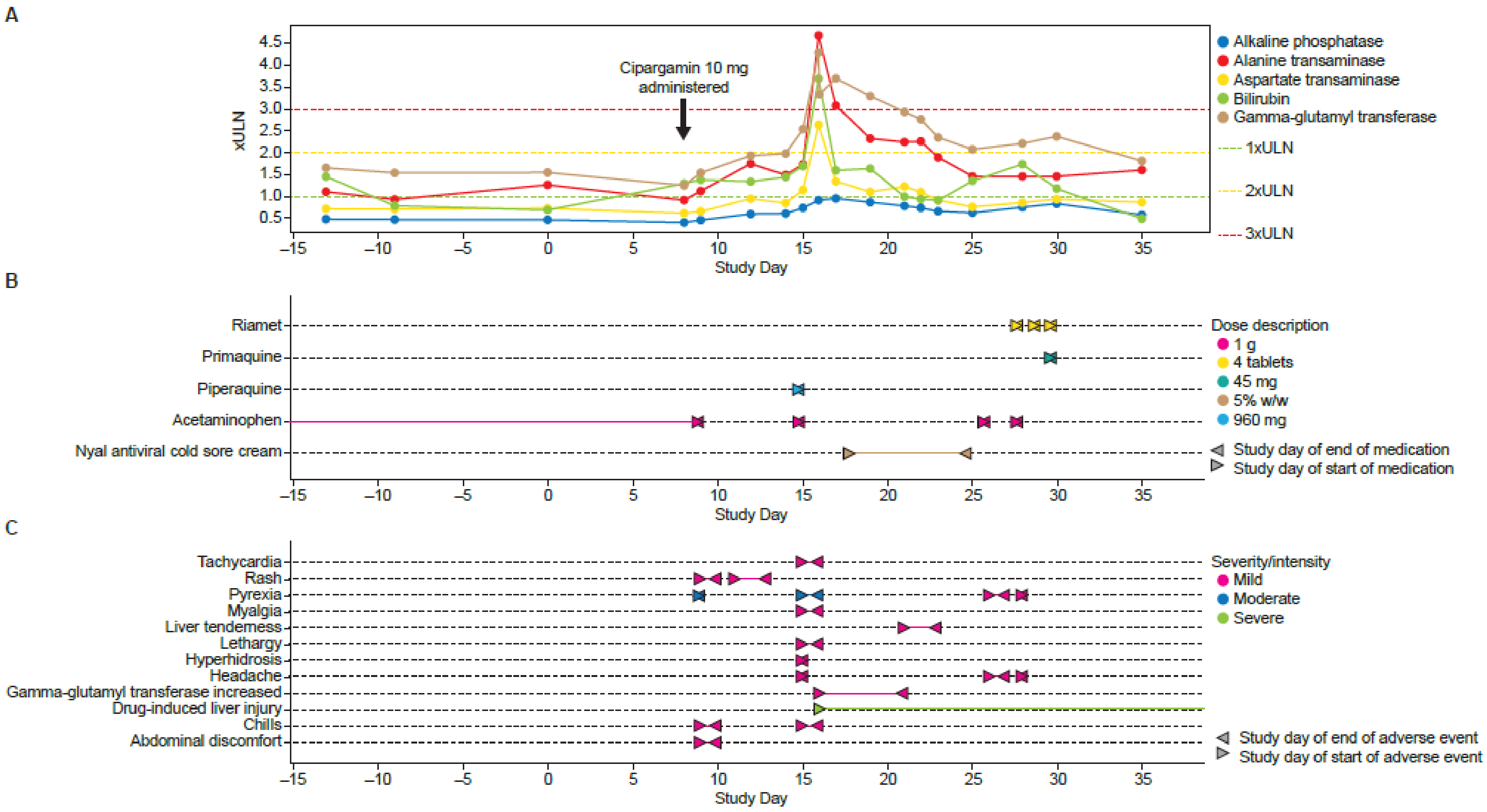

Supplemental Figure 2. Temporal relationship between miR122 and ALT levels in subjects with increased ALT levels in study QP15C01.
Cipargamin was administered on Day 8 at a sub-therapeutic dose and piperaquine on Day 11 in subject 5101, Day 15 in subject 5103 and Day 14 in subject 5108 at a therapeutic dose.

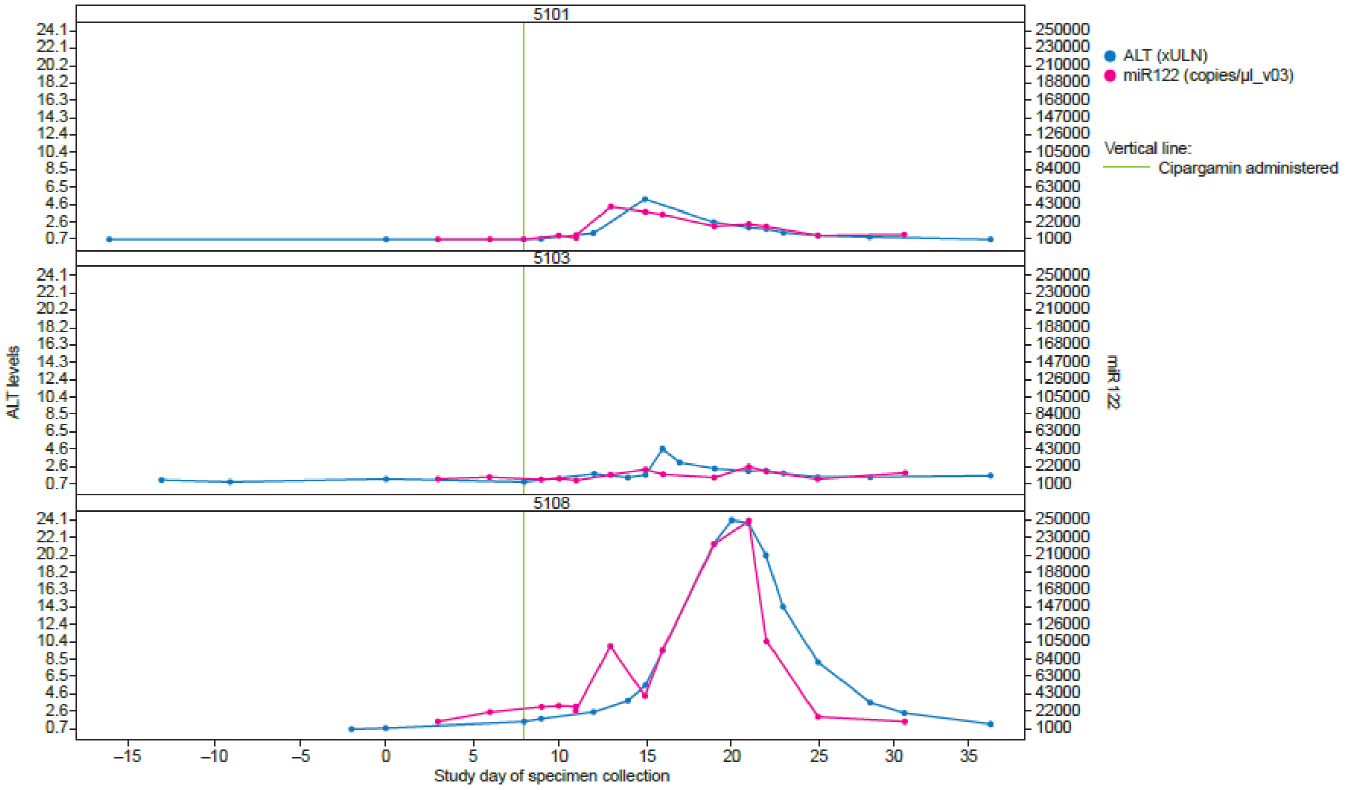

Supplemental Table 2. Acetaminophen administration to subjects with moderate (Grade 2) or severe (Grade 3 or 4) increases in ALT in IBSM studies.

Severity*	Study: Subject	Cumulative acetaminophen dose (mg)
Grade 2<br>2.5–5xULN (moderate)	QP13C05: R019	3000
	QP14C02: 007	4000
	QP14C12: R204	4000
	QP14C12: R103	0
	QP15C01: 5103	3000
	QP15C05: R109	0
	QP15C20: R107	1000
	QP15C20: R208	0
	QP16C04: R503	0
	QP16C04: R505	0
Grade 3<br>5.1–10xULN (severe)	QP14C12: R201	2000
	QP15C01: 5101	0
	QP15C05: R110	2000
	QP15C20: R102	0
Grade 4<br>>10xULN (severe)	QP13C14: R101(A)	0
	QP13C14: R104	6250
	QP13C14: R105	7000
	QP15C01: 5108	7000
	QP15C20: R101(G)	8500
	QP16C04: R502	0

*Grades are based on the World Health Organization Adverse Event Grading System.

Supplemental Figure 3. Temporal relationship between acetaminophen dosing and presence of adducts in study QP15C01.
Cipargamin was administered on Day 8 at a sub-therapeutic dose. Subjects 5101, 5103 and 5108 had moderate-to-severe increases in ALT.

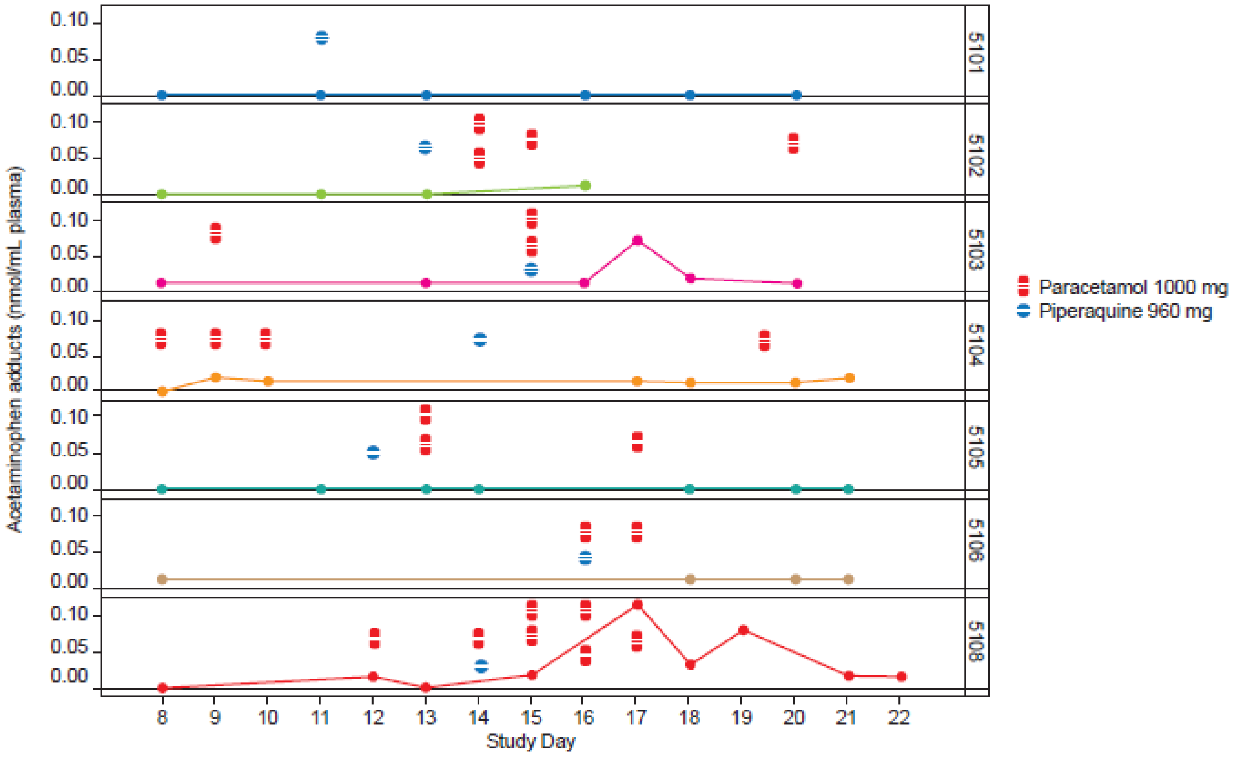

Supplemental Table 3. Data from a study of pafuramidine in the *Anopheles*-bite sporozoite challenge model. A) Risk of increased aminotransferase levels in subjects with or without malaria treated with pafuramidine and/or acetaminophen. B) Severity of changes in liver system laboratory measures with pafuramidine or placebo. Adapted from Nyunt MM, et al. Am J Trop Med Hyg 80:528-535.

A)

**Risk factors**	**Increased ALT/AST (n=10)**	**No increase in ALT/AST (n=9)**	**Risk**
No malaria, acetaminophen or pafuramidine	0	1	0
Pafuramidine alone	0	3	0
Malaria + acetaminophen	3	1	0.75
Malaria + pafuramidine	1	0	1.0
Malaria + acetaminophen + pafuramidine	6	4	0.6

B)

**Increase in:**	**Severity***	**Placebo (n=5)**	**Pafuramidine**	
			**Day –8 (n=7)**	**Day –1 (n=7)**
ALT/AST	Grade 1	1	1	2
	Grade 2	1	1	0
	Grade 3	1	1	2
	Total	3	3	4
ALP	Grade 1	1	1	0
Total bilirubin	Grade 1	1	0	2

ALT, alanine aminotransferase; AST, aspartate aminotransferase; ALP, alkaline phosphatase. *Grades are based on the World Health Organization Adverse Event Grading System.
